# Ordinal Spectrum: Mapping Ordinal Patterns into Frequency Domain

**DOI:** 10.3390/e27101027

**Published:** 2025-09-30

**Authors:** Mario Chavez, Johann H. Martínez

**Affiliations:** 1 CNRS UMR-7225, Hôpital de la Salpêtrière, 75013 Paris, France; neurodynamicslab@gmail.com; 2 Complex Systems Group and G.I.S.C, Universidad Rey Juan Carlos, 28933 Móstoles, Madrid, Spain

**Keywords:** time series, chaotic dynamics, nonlinear dynamics, symbolic dynamics

## Abstract

Classical spectral analysis characterizes linear systems effectively but often fails to reveal the nonlinear temporal structure of chaotic dynamics. We introduce the *ordinal spectrum*, a frequency-domain characterization derived from the ordinal-pattern representation of a time series. Applied to both synthetic and real-world datasets—including periodic, stochastic, and chaotic signals from physical, biological, and astronomical sources—the ordinal spectrum identifies the temporal scales implied in a possible chaotic behavior. By providing an interpretable, data-driven view of symbolic dynamics in the frequency domain, this approach complements state–space reconstructions and enhances the detection of nonlinear temporal organization that classical spectra may obscure. Its ability to distinguish between qualitatively different dynamics make it a useful tool for exploring complex time series across diverse scientific domains.

## 1. Introduction

Observed time series from a large number of physical processes generally display erratic temporal behavior. In the last decades, various measures of complexity have been proposed to characterize these data and to distinguish regular (e.g., periodic), chaotic, and random dynamics [1]. A spectral representation based on the Fourier transform is a natural way to describe oscillatory behavior in terms of frequency, magnitude, and phase. Indeed, changes in power spectra of some dynamical systems as they bifurcate to chaos have been documented [2,3,4]. However, although several measures have been proposed to quantify such spectral changes [2,5,6,7], a close relation between these measures and attractor topology remains unclear and is not supported by numerical simulations [2,7]. These open issues already suggest that spectral descriptors alone may miss essential dynamical structure.

This limitation becomes most apparent when distinct mechanisms yield look-alike spectra. It is generally accepted that time series observed from chaotic systems exhibit characteristic signatures such as fractal geometry and broadband frequency content. In particular, time series from chaotic systems often display an exponential decay in their power spectrum at high frequencies, different from the classical power–law behavior of colored stochastic noise [5,8,9]. Nevertheless, some studies have shown that spectra of colored noises cannot be related to the dynamical route to chaos [10,11]. In fact, the power spectrum estimated from chaotic sequences can be replicated by a monotonic nonlinear transformation of linearly filtered noise [12,13,14]. Taken together, this evidence indicates that classical spectral analysis alone does not provide adequate information for identifying chaotic systems.

This motivates moving beyond linear spectra toward statistics that capture phase coupling and nonlinear interactions. Although classical spectral analysis is adequate for characterizing linear systems, power spectra cannot reflect the nonlinear interactions between Fourier components of a chaotic motion [3,15,16]. Bispectral techniques have been used to investigate such nonlinear interactions [15,16], but several works have shown that higher-order statistics are required for a more complete characterization of chaotic dynamics [16,17]. More recently, other nonlinear spectral methods have been proposed to distinguish deterministic from stochastic dynamics in finite time series. The so-called symbol spectrum test developed in Refs. [18,19] does not take into account the temporal dynamics of the symbols, but rather the variability of their distribution in the symbolic sequence. The spectrum proposed in Ref. [20] characterizes, in the frequency domain, the recurrence of a reconstructed trajectory in phase space.

A complementary avenue is to characterize organization directly in state–space and via symbolization. Based on the concept of state–space reconstruction, measures such as entropies, Lyapunov exponents, and fractal dimensions have proved effective to characterize dynamics and, in favorable cases, to reconstruct equations of motion when the underlying model is deterministic [1,21]. Alternative nonlinear mapping functions have been proposed to better capture the disorder of a time series through symbolization procedures [22,23]. Among these, the method known as ordinal patterns (OP) transforms local order relations among data values and provides a robust estimation of the associated probability distribution function [24]. This ordinal representation has offered a reliable tool to discriminate, in the time domain, different dynamical regimes in time series [25,26,27]. Coarse-graining approaches [28,29], as well as the use of ordinal structures for different time delays [30], have been proposed to characterize complexity at different temporal scales. These properties make ordinal information a natural candidate to inform a frequency–domain analysis.

Motivated by these considerations, we introduce a frequency–domain tool that explicitly leverages ordinal structure. In this study we introduce the *ordinal spectrum* of time series. The method is based on a spectral transformation of a symbolic representation of the data; it relies on ordinal patterns and is therefore fully data-driven. In contrast with other nonlinear approaches, the proposed analysis provides a characterization of the data’s complexity in the frequency domain, by identifying the temporal scales implied in a chaotic dynamics. We assess the reliability of the method in distinguishing periodic or random time series from chaotic data, and we evaluate its performance on synthetic and real signals spanning linear, nonlinear, stochastic, and deterministic dynamics. Results suggest a method that may provide new insights into the non-linear oscillations observed in different real data.

## 2. Materials and Methods

**Figure 1:**
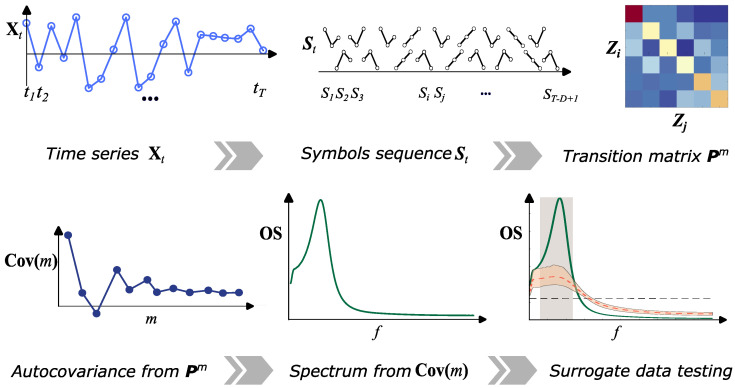
Workflow for computing the ordinal spectrum. Top: From a time series $X_{t}$ of length *T*, ordinal patterns with, e.g., embedding dimension *D* and delay τ=m=1 are constructed to produce the symbolic sequence $S_{t}$ over the D! permutations (state space Z). A first-order transition matrix $p_{ij}^{m=1}$ is then estimated from consecutive symbols. Bottom: The rank autocovariance Cov(m) is computed across lags *m* and its spectral representation yields the ordinal spectrum OS(f) (Equation (3)). Comparison with IAAFT surrogate spectra highlights the frequencies where OS(f) significantly deviates from those values obtained with linear, correlation-preserving dynamics.

The main steps to estimate the ordinal spectrum from a time series $X_{t}$ are summarized in Figure 1 and detailed in Box 1; the procedure is as follows.

*(i) Ordinal pattern representation of the data*. A symbolization procedures maps a time series $X_{t}$ onto a discretized sequence of symbols by extracting information from its amplitudes [31]. Among several symbolization proposals [32], we consider here the dynamical transformation of ordinal patterns (OP) [24]. This method maps a time series $X_{t}$ with t=1,…,T to a finite number of patterns that encode the relative amplitudes observed in the *D*-dimensional vectors $X_{t}=\left\{X_{t},X_{t+\tau },\ldots ,X_{t+(D-1)\tau }\right\}$. The elements of $X_{t}$ are mapped uniquely onto the permutation $\pi =(\pi_{0},\pi_{1},\ldots ,\pi_{D-1})$ of (0,1,…,D−1) that fulfills $X_{t+\pi_{0}\tau }⩽X_{t+\pi_{1}\tau }⩽\ldots ⩽X_{t+\pi_{D-1}\tau }$.

Box 1 **Ordinal spectrum: six-step procedure.**

**Steps**
(1) Choose embedding parameters (D,τ) and form $X_{t}=\left\{X_{t},X_{t+\tau },\ldots ,X_{t+(D-1)\tau }\right\}$ for t=(D−1)τ+1,…,T.(2) Map each $X_{t}$ to its permutation π and obtain the symbolic sequence $S_{t}$ over the D! ordinal patterns (Z).(3) Estimate the stationary probabilities $p_{i}^{*}$ and the *m*-step transition probabilities $p_{ij}^{m}$ from $S_{t}$.(4) Compute the rank autocovariance Cov(m) using Equations (1)–(2).(5) Obtain the ordinal spectrum OS(f) from Cov(m) via Equation (3).(6) Assess significance by comparing OS(f) with IAAFT-surrogate spectra $\left\{{\mathrm{OS}}^{s}\left(f\right)\right\}$ and corrected for multiple comparisons.

The set of all possible ordinal patterns derived from a time series, representing the whole embedding state space, is denoted by Z and has cardinality at most D! The entire sequence of OP extracted from $X_{t}$ is the symbol sequence $S_{t}$ of the series. Higher *D* captures more information from the data and increases the number of possible symbols. To sample the empirical distribution of ordinal patterns densely enough for a reliable probability estimation, we follow [31] and require T⩾(D+1)! The OP symbolization has several practical advantages [25,26]: (a) it is computationally efficient; (b) it is fully data-driven, with no assumptions about data ranges to define partitions; (c) it is invariant to monotonic transformations; and (d) small *D* is generally useful in descriptive analysis [24,31]. Moreover, this representation is relatively robust to noise and useful for weakly stationary series [25,31,33,34].

Unlike phase-space reconstruction, in ordinal time-series analysis the choice of the embedding dimension *D* is primarily dictated by computational cost and statistical significance given the available *T*. The selection of the embedding delay τ may influence the analysis of correlated data. To mitigate correlation effects, we select τ as either the first minimum or the zero crossing of the sample autocorrelation function ρ of $X_{t}$ (the folding time ρ=1/e is used in case of monotonic ρ).

*(ii) Capturing information dynamics from the symbolic sequence*. To characterize the time evolution of $S_{t}$ we model it as a homogeneous, ergodic Markov chain with finite state space $Z=\{z_{1},\ldots ,z_{n}\}$ (the set of distinct available permutations). Let $P^{m}=\left\{p_{ij}^{m}\right\}$ be the set of transition matrices describing the probability of leaving symbol $z_{i}$ and entering symbol $z_{j}$ at distance *m*, i.e., $p_{ij}^{m}=p\left(S_{t+m}=z_{j}|S_{t}=z_{i}\right)$. If the chain is stationary, $p\left(S_{t}=z_{i}\right)=p_{i}^{*}$, where $p_{i}^{*}$ is the invariant distribution satisfying $p_{j}^{*}=\sum_{i}p_{i}^{*}p_{ij}$.

*(iii) Characterization of symbolic dynamics at different time lags*. For a first-order Markov chain, each symbol in $S_{t}$ is conditionally independent of all but the immediately previous symbol. However, by construction, each ordinal pattern in $S_{t}$ depends on its predecessors, which induces nonzero correlation between $S_{t}$ and $S_{t+m}$ even for m>1. The correlation between $S_{t}$ and $S_{t+m}$ is termed the autocovariance at lag *m* and, for a Markov chain converging to a unique stationary distribution, it is expected to decrease as *m* increases [35,36].

We note that, in contrast with numeric signals, symbolic sets have no algebraic structure and algebraic operations are not usually meaningful. To address this, several rules have been proposed to map a symbolic sequence into a numerical domain [37,38]. The numerical algorithm used in the OP transformation yields an enumeration of permutations such that each unique ordinal pattern can be associated with a nonnegative integer, $z_{i}=i$ with i∈{1,…,D!} [39,40]. This enumeration induces a natural order of symbols: the pattern (1,…,D) and (D,…,1) are at maximal distance, representing opposite monotonic behaviors [40].

On the basis of this representation of patterns as ordered symbols, a rank variance and a rank autocovariance of the Markov chain can be obtained as follows [35,36]:

(1) $$\begin{array}{cc} \mathrm{Var}(S) & =\sum_{i}i^{2}p\left(S_{t}=z_{i}\right)-E{\left\{S_{t}\right\}}^{2},\\ \mathrm{Cov}(m) & =\mathrm{Cov}(S_{t},S_{t+m})\\ \; & =\sum_{i}\sum_{j}ijp\left(S_{t}=z_{i},S_{t+m}=z_{j}\right)-E{\left\{S_{t}\right\}}^{2}, \end{array}$$

where $E\left\{S_{t}\right\}=\sum_{i}i\;p\left(S_{t}=z_{i}\right)$. We can also write $p\left(S_{t}=z_{i},S_{t+m}=z_{j}\right)=p_{i}^{*}\;p_{ij}^{m}$, which yields:

(2) $$\mathrm{Cov}\left(m\right)=\sum_{i}\sum_{j}ij\;p_{i}^{*}\;p_{ij}^{m}-{\left(\sum_{i}i\;p_{i}^{*}\right)}^{2}.$$

*(iv) Estimation of the ordinal spectrum*. To explore the spectral properties of the ordinal-pattern sequence, the *ordinal spectrum* (OS) is obtained from the spectral representation of the autocovariance function defined above [41]:

(3) $$\mathrm{OS}\left(f\right)=\sum_{m=-(N-1)}^{N-1}\mathrm{Cov}\left(m\right)\;\exp\left(-i\;2\pi fm\right)\;.$$

Periodicity in time series yields periodic structure in the symbolic sequence and is reflected in an ordinal spectrum with clear peaks. Similarly to its classical counterpart, a random symbolic sequence is decorrelated and exhibits an approximately flat spectrum. The structure of symbolic sequences depends on the temporal correlations of the original time series [42]; consequently, their symbolic autocovariance and ordinal spectrum are expected to depend on the degree of such correlations. We notice that alternative estimators (e.g., an autoregressive model) can be used to obtain the OS(f) from $\mathrm{Cov}(S_{t},S_{t+m})$, depending on the expected bias, variance or spectral resolution [42].

*(v) Detection of relevant scales in the ordinal spectrum*. Peaks in the ordinal spectrum could arise from large autocorrelation at certain time lags in the original symbolic sequence $S_{t}$. To rule out this possibility, we compare the ordinal spectrum with those obtained from an ensemble $\left\{X_{t}^{s}\right\}$ of surrogate series that replicate the linear autocorrelation and amplitude distribution of $X_{t}$. We use Iterative Amplitude Adjusted Fourier Transform (IAAFT) surrogates [13,14], which preserve the autocorrelation function and amplitude distribution while destroying higher-order structure. For each $X_{t}^{s}$, we repeat steps *(i)–(iv)* to compute a set $\left\{{\mathrm{OS}}^{s}\left(f\right)\right\}$. If any value in OS(f) is statistically distant from the distribution of $\left\{{\mathrm{OS}}^{s}\left(f\right)\right\}$, we reject the null hypothesis of a linear stochastic process. The statistical significance is assessed by a z-test to quantify the statistical deviation from those values obtained in the ensemble of surrogate data (set here to N=500). All significance tests are set at p<0.05 with a correction for multiple comparisons (False Discovery Rate [43]) over frequencies *f*.

## 3. Results

To demonstrate our method, we apply it first to synthetic data generated by a logistic map, defined by the iterative equation $x_{n+1}=rx_{n}\left(1-x_{n}\right)$ where *r* is the bifurcation parameter. This nonlinear map has several transitions in the dynamics occurring during $r\in \left[3,4\right)$, with several period-doubling cascades before the onset of chaos at r≈3.56995 [44]. Beyond this value, several isolated ranges of *r* display non-chaotic behavior [44]. For this analysis, each time series consists of T=2000 points, after discarding the first 1000 iterations to eliminate transient effects.

**Figure 2:**
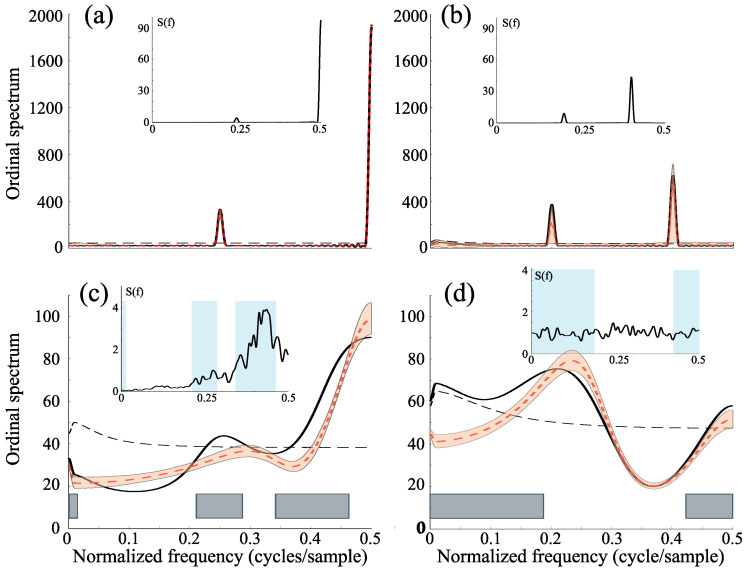
Ordinal spectra as a function of normalized frequency (black curves) for time series generated by the logistic map with embedding parameters D=3 and τ=1: (**a**) periodic dynamics at r=3.55; (**b**) periodic dynamics at r=3.739; (**c**) chaotic dynamics at r=3.8; and (**d**) chaotic dynamics at r=4. Gray boxes in the main panels highlight frequency regions where the ordinal spectra differ significantly from those obtained with surrogate data. Slashed black curves represent the average spectra from randomized symbolic sequences (not from IAAFT surrogates). Orange shaded regions and inner slashed red curves denote the 5th–95th percentiles and the average spectra across IAAFT surrogates, respectively. Insets show the power spectra of the original time series (normalized frequency), with blue shading the frequency bands identified in OS(f).

Main plots in Figure 2a,b show that, despite the large peaks observed in the ordinal spectrum, the dynamical properties of the periodic process are not statistically different from those replicated by the surrogate data and thus, the null hypothesis cannot be rejected at any frequency. As expected for chaotic sequences, results in Figure 2c,d indicate that the ordinal spectrum identifies frequency ranges in which the dynamics significantly deviate from the surrogate data, thereby revealing signatures of increased dynamical complexity.

We also test our method on data generated by the Rössler system whose equations are given by $\left[\dot{x}=-y-z,\;\dot{y}=x+ay,\;\dot{z}=2+z\left(x-4\right)\right]$, with control parameter a∈[0.25,0.55]. Similar to the logistic map, Rössler system has several periodic transitions before the onset of chaos at a≈0.385. For larger values of *a*, some periodic windows can still be observed. Each time series has length $T=10^{4}$, with the first 1000 samples discarded to remove transients.

**Figure 3:**
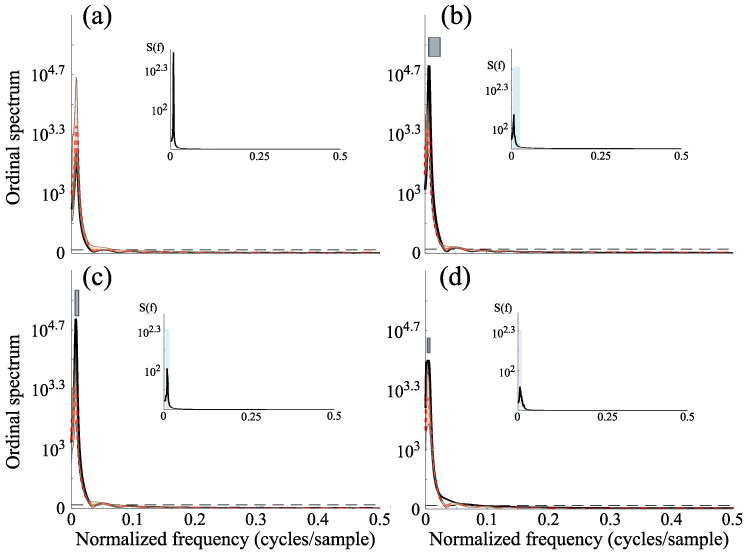
Ordinal spectra against normalized frequency for data generated from the *x*-component of the Rössler system (D=4 and τ=30 samples) with: periodic behaviors (**a**) a=0.30; and chaotic dynamics (**b**) a=0.42; and (**c**) a=0.54. (**d**) Chaotic regime as in (**c**) but truncated to ∼10 oscillation cycles. Same stipulations as in the caption of Figure 2.

Figure 3a–c demonstrate that the ordinal spectrum test discriminates between periodic and chaotic regimes. In the periodic cases, the spectra of the original time series are statistically indistinguishable from those of the surrogates, and the null hypothesis cannot be rejected. In contrast, in the chaotic regime, the ordinal spectrum reveals a frequency interval where the dynamics deviate significantly from the surrogate data, indicating the presence of nonlinear correlations not explained by linearly filtered noise. Notably, this distinction is not apparent in the power spectra, which show similar power-law decays for both original and surrogate series.

We also assess the sensitivity of the method to time series length. As shown in Figure 3d, chaos in the logistic map is consistently detected when T≥500. For the chaotic R"ossler system, the method requires time series longer than approximately ten times the fundamental oscillation period to reliably reject the null hypothesis. For shorter sequences, no statistically significant differences from surrogate data are detected, and the method fails to identify chaos.

In addition to testing across deterministic time series (chaotic or not), we evaluated the method on non-chaotic stochastic data. Here we firstly evaluated a Gaussian noise with distribution N(0,1) and a stochastic process with a power law spectrum $S\left(f\right)\propto f^{-\alpha }$ where α=1. For each stochastic process, the ordinal spectrum is computed using time series of length T=2000.

**Figure 4:**
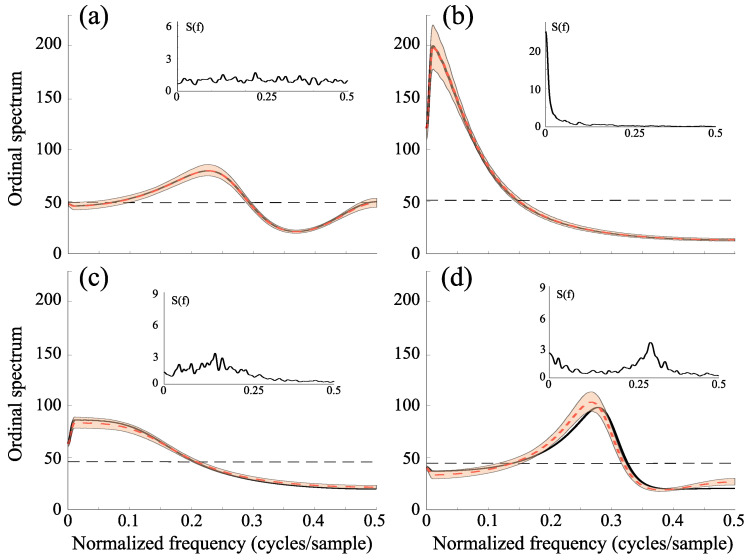
Ordinal spectra for stochastic data generated from: (**a**) a Gaussian distribution (D=4 and τ=1 sample); (**b**) Power law noise (D=4 and τ=4 samples); (**c**) nonlinear system driven by non-Gaussian noise (D=4 and τ=2 samples); and (**d**) non-monotonic nonlinear transformation of a linearly filtered noise (D=4 and τ=3 samples). The same conventions as in Figure 2 are used.

The results in Figure 4a,b show that, although the ordinal spectra reflect the temporal correlations of the original series, they are statistically indistinguishable from those obtained from surrogate data. Consistently, the statistical tests do not reject the null hypothesis for either white (uncorrelated) or colored (correlated) noise.

We then analyzed nonlinear systems driven by non-Gaussian noise. The first system was defined as $x_{t}=0.5x_{t-1}-0.3x_{t-2}+0.1y_{t-2}+0.1x_{t-2}^{2}+0.4y_{t-1}^{2}+0.0025\eta_{t}^{\prime }$ and $y_{t}=\sin\left(4\pi t\right)+\sin\left(6\pi t\right)+0.0025\eta_{t}^{\prime \prime }$, where noises $\{\eta_{t}^{\prime }$,$\eta_{t}^{\prime \prime }\}$ are iid drawn from the Laplacian distribution $p\left(\eta \right)=\frac{1}{4b}\exp\left(\frac{-|\eta -\mu |}{b}\right)$, with μ=0 and b=1. To further evaluate performance under the null hypothesis of nonlinearly transformed stochastic processes, we considered a static nonlinear and non-monotonic transformation $x_{t}=\tanh^{2}\left(y_{t}\right)$ applied to the linear non-Gaussian process $y_{t}=0.4y_{t-3}-0.3y_{t-2}0.2y_{t-1}+e_{t}$, where $e_{t}$ is obtained by squaring a uniform noise with amplitude distribution between −0.5 and 0.5 [45]. For these two models, the data length is also set to T=2000, after discarding the first 1000 points.

A clear distinction between chaos and stochastic behavior can be difficult for data generated by nonlinear systems driven by non-Gaussian noises [13,14]. Similarly, it is well known that nonlinear transformations may introduce sufficient phase correlations in linearly filtered noises making difficult to identify the stochastic behavior. Results depicted in Figure 4c,d indicate that the ordinal spectrum, in combination with the IAAFT algorithm, correctly diagnoses the nonlinear and non-Gaussian models as stochastic process, including the static non-monotonic transformation of a non Gaussian random process.

These findings highlight three key points: *(i)* large peaks in the ordinal spectrum, by themselves, cannot be taken as evidence of chaotic dynamics; *(ii)* for chaotic time series, the frequency intervals in which the ordinal spectrum significantly deviates from that of surrogate data do not necessarily span the entire frequency range; and *(iii)* regardless of whether the underlying dynamics are periodic or chaotic, random shuffling of the symbolic sequence invariably yields a flat spectrum. Consequently, statistical tests based solely on shuffled symbolic sequences are insufficient to distinguish chaos from stochastic processes.

Finally, we demonstrate the potentials of our method on real data of different nature: epidemiology (measles and cholera time series [46]), astrophysics (the sunspots number series) and neuroscience (electroencephalographic data from an epileptic patient). Since the data sets have different lengths, we apply the ordinal symbolic transformation in different embedding dimensions, following the condition T⩾(D+1)! [26].

**Figure 5:**
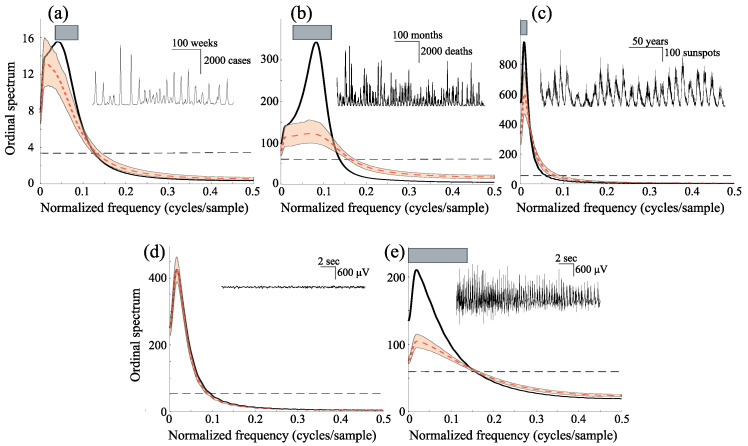
Ordinal spectra (black curves) of real-world data. Insets display the original time series with their respective temporal and amplitude scales. (**a**) Measles cases (D=3, τ=4); (**b**) cholera data (D=4, τ=2); (**c**) sunspot numbers (D=4, τ=30); (**d**) interictal EEG; and (**e**) EEG during an epileptic seizure (D=4, τ=5). Slashed curves represent the average spectra from randomly shuffled symbolic sequences. Gray boxes in the main panels indicate frequency ranges where the ordinal spectra differ significantly from those of surrogate data. The same conventions as in Figure 2 are used.

The pattern of measles epidemics in several countries is among the best documented population cycles in ecology. Different studies have proposed evidence for low dimensional dynamics in epidemiological time series [47,48]. The inset in Figure 5a shows the monthly cases of measles in Copenhague, Denmark, between 1927 and 1968 [48]. For this series, our method clearly rejects the null hypothesis of a stochastic process. Consistent with previous findings, the results indicate that measles dynamics cannot be adequately described by conventional linear models, and instead reflect a low-dimensional nonlinear structure.

Interannual cycles in many infectious diseases arise from the interplay between intrinsic and extrinsic factors [49]. These interactions can generate oscillatory patterns of considerable complexity, including chaos [50]. The inset in Figure 5b depicts the monthly deaths from cholera in Dacca, East Bengal between 1891 and 1940 [51,52]. Our analysis indicates that the ordinal spectrum captures nonlinear deterministic features in the data, suggesting the presence of low-dimensional dynamics. This result is consistent with earlier mathematical models of seasonally driven epidemics [50].

Solar activity is governed by the emergence of magnetic flux through the photosphere, forming active regions that include sunspots. While the dominant feature of solar activity is the modulated 11-year cycle [53], several studies have proposed that the irregular component of sunspot activity reflects low-dimensional chaotic dynamics [53]. The inset in Figure 5c shows the monthly mean total sunspot number (the arithmetic mean of the daily total sunspot number over all days of each calendar month) between 1749 and 2020 [54]. Our method rejects the null hypothesis of stochasticity, in agreement with previous results suggesting that sunspot fluctuations can be described by nonlinear, possibly chaotic, dynamics [53].

Electroencephalographic (EEG) signals, like many biological and medical time series, exhibit strong nonlinearities during both cognitive and pathological states [55]. In epilepsy, the dynamical properties of EEG signals can serve as markers of the epileptogenic zone [56]. We analyzed scalp EEG recordings from a patient with intractable epileptic seizures, obtained at 102.4 Hz using a right central (C4) electrode with linked-earlobe reference [57,58]. Time series ploted in Figure 5d,e correspond to data from interictal and ictal (seizure) period, respectively. These results confirm previous findings suggesting that interictal EEG dynamics can be associate to a stochastic process, whereas a low dimensional dynamics characterizes epileptic seizures [56].

## 4. Discussion

In this study, we introduced a nonlinear spectral method for characterizing complexity in the frequency domain based on ordinal patterns. Our approach provides a robust framework for distinguishing chaotic fluctuations from stochastic dynamics in finite time series. By comparing the spectral information of the symbolic representation of a time series $X_{t}$ with that of linearly filtered surrogate data, the method effectively accounts for static nonlinear transformations of linear data and yields reliable results even in the presence of correlated noise or nonlinearly transformed stochastic processes.

We demonstrated that the detection of chaotic oscillations can be successfully addressed through spectral analysis of ordinal symbolic sequences. The main advantages of the proposed method are its simplicity, reliability, and computational efficiency. Being fully data-driven, it does not require prior knowledge of the time series to construct the symbolic representation, making it highly suitable for real-world applications. Furthermore, while our approach is based on ordinal patterns, it can be readily extended to other symbolic representations and alternative spectral analyses derived from symbolic data.

Application to real-world data confirms the practical utility of the method. Our results support the presence of low-dimensional chaotic dynamics in sunspot time series and epidemiological data, such as measles and cholera cases. In neuroscience, EEG recordings during epileptic seizures exhibit complex, low-dimensional dynamics, whereas interictal activity is consistent with stochastic processes. These findings suggest that nonlinear spectral methods provide a more complete characterization of time series, revealing underlying dynamical structures that are not captured by conventional linear or power spectral analyses.

The ordinal spectrum can be particularly useful for capturing nonlinear correlations in signals, with future applications in forecasting of nonlinear time series. Even though in this study we only discuss the case for univariate data, the extension to multivariate cases is straightforward. In particular, extending the ordinal spectrum to a nonlinear cross-spectrum (or coherence) could provide a more comprehensive characterization of complex systems. The original algorithm could also be extended to investigate higher-order statistics (i.e., ordinal higher-order statistics), since its main building blocks rely on the covariance function. Such an extension would allow for the analysis of more intricate dependencies beyond second-order correlations, thereby providing deeper insights into the underlying dynamics of nonlinear systems.

Overall, the proposed approach offers a powerful tool for the analysis of complex oscillatory signals across diverse domains, including biomedical, ecological, financial, and climate systems. By combining symbolic representations with spectral analysis, it enables a deeper understanding of the intrinsic dynamics of time series and provides a rigorous framework for distinguishing chaotic oscillations.

## Data Availability

The data presented in this study are available on request from the corresponding author.
